# Metformin’s Effects on Cognitive Function from a Biovariance Perspective: A Narrative Review

**DOI:** 10.3390/ijms26041783

**Published:** 2025-02-19

**Authors:** Dimitrie Chele, Carmen-Adella Sirbu, Marian Mitrica, Mihai Toma, Octavian Vasiliu, Anca-Maria Sirbu, Francois Jerome Authier, Dan Mischianu, Alice Elena Munteanu

**Affiliations:** 1 Department of Neurology, Elias Emergency University Hospital, 011461 Bucharest, Romania; chele.dimitrie@gmail.com; 2 Clinical Neurosciences Department, University of Medicine and Pharmacy “Carol Davila” Bucharest, 050474 Bucharest, Romania; titimitrica@yahoo.com (M.M.); octavvasiliu@yahoo.com (O.V.); 3 Academy of Romanian Scientists, 050045 Bucharest, Romania; 4 Department of Medical-Surgical and Prophylactical Disciplines, Faculty of Medicine, ‘Titu Maiorescu’ University, 031593 Bucharest, Romania; iahim_t@yahoo.com (M.T.); dralicepopescu@yahoo.com (A.E.M.); 5 Department of Psychiatry, ‘Dr. Carol Davila’ Central Military Emergency University Hospital, 010825 Bucharest, Romania; 6 National Institute of Medical Expertise and Recovery of Work Capacity, Panduri 22, 050659 Bucharest, Romania; 7 Neuromuscular Reference Center, Henri Mondor University Hospital, Assistance Publique–Hôpitaux de Paris, 94000 Créteil, France; 8 INSERM U955-Team Relaix, Faculty of Health, Paris Est-Creteil University, 94010 Créteil, France; 9 Department No. 3, University of Medicine and Pharmacy “Carol Davila” Bucharest, 050474 Bucharest, Romania

**Keywords:** metformin, diabetes, cognition, neuroprotection, biovariance

## Abstract

This study examines the effects of metformin on brain functions focusing on the variability of the results reported in the literature. While some studies suggest that metformin may have neuroprotective effects in diabetic patients, others report an insignificant impact of metformin on cognitive function, or even a negative effect. We propose that this inconsistency may be due to intrinsic cellular-level variability among individuals, which we term “biovariance”. Biovariance persists even in demographically homogeneous samples due to complex and stochastic biological processes. Additionally, the complex metabolic actions of metformin, including its influence on neuroenergetics and neuronal survival, may produce different effects depending on individual metabolic characteristics.

## 1. Introduction

Metformin, a drug belonging to the biguanide class, is a widely prescribed medication for managing non-insulin-dependent type 2 diabetes (T2D) and has garnered significant scientific attention due to its multifaceted effects on cellular metabolism and its emerging therapeutic potential in diverse areas of medicine. Its well-documented mechanisms, such as enhancing insulin sensitivity, reducing hepatic gluconeogenesis, and improving overall energy metabolism, have paved the way for research into a broad range of possible therapeutic applications [1], spanning from antitumor effects to roles in neuroplasticity and cognitive function. Additionally, metformin’s therapeutic potential has been evaluated in a variety of metabolic and endocrine disorders, such as polycystic ovary syndrome [2].

This growing interest in metformin has spurred numerous studies exploring its effects across various human pathologies, including cardiovascular and metabolic diseases [3,4], inflammatory conditions [5], processes associated with senescence and anti-aging properties [6], and psychiatric conditions [7].

During the COVID-19 pandemic, observational studies reported that diabetic patients treated with metformin had a lower risk of severe complications and mortality compared to those who were either untreated or receiving alternative antidiabetic therapies [8]. The hypothesized mechanisms underlying this protective effect include metformin’s immunomodulatory properties, its ability to reduce systemic inflammation, and its influence on key biochemical signaling pathways involved in cellular defense mechanisms. These findings highlight metformin’s ability to modulate fundamental aspects of cellular functionality, including immune response regulation and metabolic signaling, further supporting its broad therapeutic potential beyond glycemic control.

This review examines the intriguing neuropsychiatric effects of metformin, with a focus on its influence on cognitive function and the underlying biocellular mechanisms that contribute to interindividual variation in its effects.

Cognitive decline is a well-recognized complication of diabetes [9], hypothesized to be driven by glycemic fluctuations, insulin resistance, and disruptions in neuronal metabolism. Increasing evidence suggests that insulin dysfunction and high glycemic variability in the central nervous system (CNS) play a pivotal role in neurodegenerative disorders, such as Alzheimer’s disease (AD), giving rise to the concept of “Type 3 diabetes” [10]. While not officially recognized as a distinct clinical entity, this concept is supported by studies demonstrating overlapping pathological features between insulin resistance, a characteristic of T2D, and Alzheimer’s disease, including amyloid-β accumulation, mitochondrial dysfunction, oxidative stress, chronic neuroinflammation, and impaired glucose metabolism in the brain [11,12].

Another class of antidiabetic drugs, glucagon-like peptide-1 (GLP-1) receptor agonists (GLP-1 RAs), have also been explored for their neuroprotective potential [13]. Substantial evidence suggests that GLP-1 RAs not only enhance insulin secretion and improve glycemic control but also exert protective effects on the CNS, primarily through their neurovascular benefits [14]. GLP-1 signaling has been shown to modulate oxidative stress, inhibit tau phosphorylation, and reduce amyloid-β accumulation [15], processes that are closely linked to Alzheimer’s disease and other neurodegenerative disorders.

Diabetes has been linked to both structural and functional alterations in brain connectivity, particularly affecting white matter integrity and synaptic plasticity, which are crucial for cognitive processing. These findings align with research suggesting that chronic hyperglycemia and insulin resistance may exacerbate neurovascular damage, impair cerebral perfusion, and accelerate neurodegeneration, thereby increasing the risk of cognitive impairment and dementia.

The studies reviewed in this article have reported mixed findings regarding metformin’s effect on cognitive function in both human and non-human subjects, with notable variations across specific population subgroups. Some reports suggest that metformin exerts neuroprotective properties, potentially mitigating diabetes-related cognitive decline by modulating inflammation, promoting neurogenesis, and enhancing insulin signaling in the brain. However, other studies find no significant association between metformin treatment and cognitive function, or even raise concerns about potential adverse effects in certain populations. These findings highlight the need for a deeper understanding of how individual differences shape the neurocognitive effects of metformin.

## 2. Scope and Approach

The present review aims to reconcile the conflicting effects of metformin on cognitive function by identifying studies reporting opposing results and proposing a possible biochemical explanation. As this is a narrative review, the selection and synthesis of relevant literature were conducted in accordance with the SANRA (Scale for the Assessment of Narrative Review Articles) guidelines [16]. The SANRA checklist is provided in the Supplementary Materials.

The documentation of studies was performed through a targeted search of the PubMed and Google Scholar databases. These sources were chosen due to their extensive indexing of biomedical research and the availability of full-text articles relevant to metformin’s neurocognitive effects. Additional literature was identified via citation tracking of key review articles and meta-analyses.

To identify relevant studies, combinations of subject-specific terms were used: “metformin”, “diabetes”, “T2D”, “white matter”, “cognition”, “dementia”, ”memory”, “inflammation”, “mechanism of action”, “neuro”, “molecular”, “cellular target”, “stroke”, and “endothelium”.

The search focused on articles published in recent years, employing truncation operators to broaden terminological coverage (e.g., “metformin”, “diabet*” “neuro*”, and “cognit*”). The selection of studies was based on the following eligibility criteria:

Inclusion criteria: Peer-reviewed studies published in English after 2018, clinical trials, relevant preclinical studies, meta-analyses, and review articles specifically investigating metformin’s impact on cognitive function, neurodegenerative disorders, or brain structural changes. Studies analyzing metformin’s molecular mechanisms of action in inflammation and neurodegeneration were also included.

Exclusion criteria: Studies not explicitly assessing metformin’s effect on brain function, studies lacking differentiation between metformin and other antidiabetic drugs, articles published in languages other than English, small observational studies without adequate control, and studies with methodological limitations (e.g., risk of bias or inadequate statistical power). Case reports and case series were excluded to maintain focus on robust experimental and clinical evidence.

**Figure 1:**
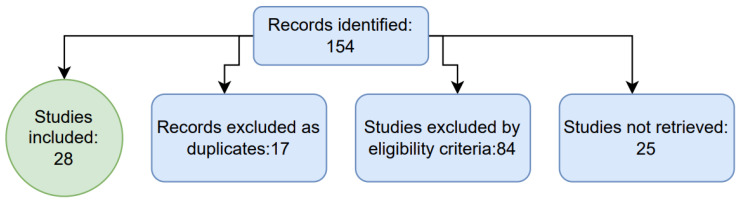
This diagram illustrates the selection and analysis process of the studies.

Relevant studies were identified through a manual screening of the search results, ensuring a thorough evaluation of their design characteristics (Figure 1). This approach allowed for a critical appraisal of inclusion and exclusion criteria while also helping to mitigate the risk of overlooking relevant studies due to database indexing limitations.

In addition to the primary articles identified through the systematic search, additional references cited within the bibliographies of selected studies were reviewed. Preclinical animal studies examining metformin’s effects on neurobiological structures and cellular metabolism were also included, as these studies offer complementary mechanistic insights to clinical data. The study assessments took into account design characteristics, statistical analysis methods, population sizes, and follow-up durations where applicable, with the primary emphasis placed on the authors’ conclusions. Table 1 provides an overview of the key studies exploring the impact of metformin.

## 3. Effects of Metformin on Metabolism and Cognition

Metformin is a first-line drug used in the treatment of type 2 diabetes. Its hypoglycemic effect is primarily attributed to a reduction in endogenous glucose production at the hepatic level by inhibiting gluconeogenesis [43]. However, other mechanisms have also been well documented, such as the improvement of insulin sensitivity in peripheral tissues, which promotes glucose utilization by muscles and other tissues. Among the proposed molecular mechanisms are the stimulation of cellular expression of the insulin-sensitive glucose transporter GLUT4 and the inhibition of SHIP2 enzyme activity [44,45,46,47]. The complete basis for the hypoglycemic effect of metformin has yet to be fully elucidated.

The bioavailability of metformin is relatively modest, ranging between 50 and 60% after oral administration [48]. Its absorption occurs primarily in the small intestine, is influenced by diet [49], and the drug is largely excreted unchanged by the kidneys. An essential characteristic of metformin is that it does not bind to plasma proteins and is not metabolized by the liver. Metformin is widely distributed throughout body tissues. Furthermore, the unabsorbed portion of metformin (approximately 50%) can influence the intestinal microflora, with evidence showing that metformin affects the microbiome [50,51,52]. One study even suggests a possible correlation between an individual’s intestinal microbiome and their response to metformin [53].

Regarding metformin’s action on the brain, it is important to note that animal studies confirm its ability to cross the blood–brain barrier [54,55]. We emphasize that the permeability of the blood–brain barrier varies between species and, additionally, between individuals. Metformin concentrations in cerebrospinal fluid (CSF) are lower than in plasma, and studies measuring CSF concentrations in human subjects following oral administration are limited and address this issue only indirectly.

Metformin can influence cognitive function both directly and indirectly.

Metformin exerts multiple metabolic effects that may indirectly enhance cognitive function by optimizing metabolic processes (such as glucose homeostasis, lipid metabolism, and insulin sensitivity) and reducing cardiovascular risk factors. Chronic hyperglycemia is a major risk factor for cognitive decline and structural brain damage [56]. Numerous studies correlate diabetes with an increased risk of developing dementia or other cognitive disorders [9,57,58,59]. It should be noted that beyond the impact of glycemic fluctuations, diabetes is associated with other metabolic dysfunctions that negatively affect the brain. Additionally, metformin exhibits significant anti-inflammatory and antioxidant effects [5,60,61,62], which may partially counteract the oxidative stress and inflammation associated with high glycemic variability.

Diabetes is also a significant cardiovascular risk factor [63,64], and metformin has demonstrated a protective role on cardiovascular health as well as on vascular endothelium [65,66,67], which may contribute to its indirect effects on cognition.

Beyond its metabolic and cardiovascular effects, metformin is also known to have direct neuroprotective actions. Evidence suggests that metformin activates the 5′ adenosine monophosphate-activated protein kinase (AMPK) pathway [68], which regulates cellular energy homeostasis. AMPK activation in brain neurons can promote neurogenesis, reduce inflammation, and improve mitochondrial function, all of which are important for cognitive health.

Numerous studies have demonstrated the beneficial effects of metformin on cognitive function in patients with type 2 diabetes (T2D), suggesting improvements in short-term memory, a reduced risk of developing dementia, and enhanced executive function [17,18,19,22,27,28,29,30,31]. One study identified interesting correlations between metformin, the gut microbiome, and the risk of developing dementia [21].

Regarding its multimodal impact on cognitive function, a recent animal study found a positive correlation between metformin and cognitive function (as measured by attention and inhibitory control) in young subjects [69], which shifted to a negative correlation in older subjects, suggesting an age-related effect on cognitive performance. Notably, two human studies reported more pronounced effects of metformin in Western populations [19,29], thereby raising the hypothesis that racial factors may influence the effects of metformin.

Interestingly, positive cognitive effects were also observed in patients with neuropsychiatric conditions. One study reported improvements in cognitive function and psychotic symptoms in patients with schizophrenia [36]. Similarly, studies indicate a possible anti-epileptic effect of metformin, suggesting it may modulate multiple factors implicated in the etiopathogenesis of epilepsy [37,38].

However, some research has suggested that the cognitive benefits of metformin may be time-limited, with a tendency to diminish or even reverse with long-term use. Additionally, some studies have shown no cognitive benefits from metformin treatment, including in the prevention of dementia [25,32], and others have even suggested an increased risk of AD associated with metformin use [39]. Furthermore, one study found a dose-related correlation, with lower doses linked to a reduced risk of developing dementia, while higher doses showed no benefit [26]. A similar correlation was observed in another study [70], led by the same primary author, specifically in the context of Parkinson’s disease.

Regarding other neurodegenerative conditions, the results remain inconclusive. Some studies did not find any correlation between metformin use and the risk of developing Parkinson’s disease [24], while one study suggested a possible association between metformin and an increased risk of Parkinson’s disease [34]. This finding partially contradicts other studies that suggest metformin may reduce the incidence and severity of neurodegenerative diseases [19,20]. The conflicting findings on metformin’s impact on Parkinson’s disease have been investigated in several studies [71], none of which have demonstrated a definitive effect. Interestingly, one study in animal models of Parkinson’s disease demonstrated a positive impact on motor function, potentially mediated by metformin’s effects on astrocytes [72].

Several studies have reported that metformin has a positive impact on cerebral white matter in diabetic individuals, helping to preserve white matter microarchitecture [20]. Long-term metformin use has been associated with increased white matter integrity and reduced diabetes-related brain volume loss in metformin-treated patients [23], particularly in regions, such as the parietal lobes and cingulate cortex, which are involved in spatial orientation and memory processes. Moreover, a study based on fractional anisotropy measurements showed increased cerebral parenchymal integrity in diabetic patients treated with metformin [20].

In a murine model, one study hypothesized that metformin stimulates oligodendrocyte progenitor cells (OPCs) under hypoxic conditions [26]. Another animal study demonstrated metformin’s anti-inflammatory and neuroprotective properties against the neurotoxic effects of streptozotocin [33], which is a compound used to mimic Alzheimer’s dementia in animal experiments. Additionally, metformin administration counteracted neurotoxic effects from repeated alcohol exposure, indicating a neuroprotective potential under toxic conditions [35].

The hypothesis that metformin acts as a cardiovascular protective factor has also been explored, and several studies have demonstrated its beneficial effects on endothelial function and its role in reducing oxidative stress at this level [67,73,74]. These findings may be crucial in understanding metformin’s role in cognitive protection, particularly through the enhancement of cerebral circulation and the prevention of ischemic strokes. Similarly, several studies have reported a decrease in the incidence and severity of ischemic attacks among patients already undergoing treatment with metformin, as well as a positive association between metformin use and improved clinical outcomes [40,41,42].

Additionally, studies aimed at elucidating metformin’s mechanism of action at the biocellular level were identified. The primary cellular targets of metformin are illustrated in the Table 2. Although other cellular targets are not included due to challenges in defining metformin’s specific effects on them, these targets certainly play a role in explaining and understanding metformin’s mechanism of action.

## 4. Biovariance: A New Frontier in Personalized Medicine

Interindividual variability is a well-documented concept in medical sciences, explaining differences in response to a particular treatment between individuals [83]. In another sense, this reflects the axiom “each individual is unique”. This variability accounts for the fact that a drug can exhibit a different action profile from one patient to another and may also cause adverse effects or allergic reactions in certain individuals. Clinical trials primarily assess general effects within a population, allowing conclusions to be drawn about efficacy, optimal dosages, and safety profiles [84]. Ultimately, a risk–benefit ratio is calculated, determining if the drug qualifies for therapeutic applications.

Interindividual variability arises from numerous factors, ranging from age, genetics, and environmental influences, to the intricate dynamics of chemical reactions within cells. Unlike other factors, cellular factors are challenging to use as selection filters due to their stochastic and partly unpredictable nature, as evidenced by studies that have explored this aspect [85,86]. Biovariance addresses this type of variability, which cannot be eliminated by selection criteria but can be reduced if the criteria are adapted to target metabolic function. Also, biovariance is particularly relevant in metabolic disorders like diabetes, where individual differences in insulin sensitivity, glucose metabolism, and mitochondrial function can significantly alter drug responses. This approach enables a personalized therapeutic strategy and maximizes the therapeutic potential of drugs by identifying subgroups with a strong positive response.

Biovariance refers to the heterogeneity of a drug’s effect, characterizing the response to the treatment rather than the substance itself. Its biological foundation lies in the complex dynamics of the metabolic reactions occurring at the cellular level, which are influenced by the pre-existing biochemical context, competition for receptors, and subtle molecular interactions that generate stochastic behavior. Therefore, biovariance is fundamentally tied to how living organisms process and respond to a range of substances, reflecting the complexity of the metabolic interactions that vary among individuals and even within the same individual. While biovariance is directly related to the complexity of a biological system, this relationship is not linear; as complexity increases, biovariance tends to increase exponentially. The brain is a striking example: due to the unique and highly varied organization of neuronal networks, synaptic plasticity, and the dynamic interactions between neurons, the central nervous system introduces an additional layer of variability in response to treatments. This is consistent with the findings of our study, which demonstrate the difficulty in establishing a clear effect of metformin on cognitive function based on the existing literature.

Metformin is an example of a drug with high biovariance. As we have shown, some studies suggest that metformin has a neuroprotective role, while others indicate a lack of effect or even potential cognitive risks associated with its use. This variability highlights the importance of considering individual metabolic profiles when evaluating the cognitive effects of metformin. By applying the concept of biovariance to metformin trials, future studies can refine patient selection criteria by integrating metabolic markers, in order to stratify participants more effectively. Such an approach would reduce the risk of inconclusive findings and enhance the relevance of therapeutic recommendations for diabetic patients.

### 4.1. Role of Biovariance in Shaping Clinical Trial Design

We believe that one of the most significant consequences of accepting and validating the concept of biovariance would be a reorientation of clinical trial design. This would involve adapting selection criteria to include specific markers of cellular metabolism and selecting patients based on the characteristics of their metabolome. Such an approach would enable the development of new, relevant evaluation criteria for metabolic status, providing a more accurate picture of how patients respond to treatments according to their metabolic profiles and bringing us closer to the goal of truly personalized medicine.

Most medical studies aim to identify a correlation between the administration of a substance and the occurrence of a particular effect within a population. While these studies provide valuable clinical insights, their methodology often falls short when investigating effects with high biovariance. Ignoring inherent interindividual variability can lead to misinterpretations of the data and frequently results in the conclusion that further, larger studies are necessary. Acceptance of the biovariance concept could, therefore, help halt the “race” for ever-larger, costlier studies, as the biovariant drug effects of a drug will continue to differ among individuals. Consequently, even studies with substantial sample sizes, numbering in the hundreds of thousands of participants, may still fail to yield conclusive results. In contrast, a single, highly relevant study that identifies distinct or even opposing effects within a homogeneous sample, carefully selected based on well-defined criteria, could effectively reveal the biovariant nature of the effects.

**Figure 2:**
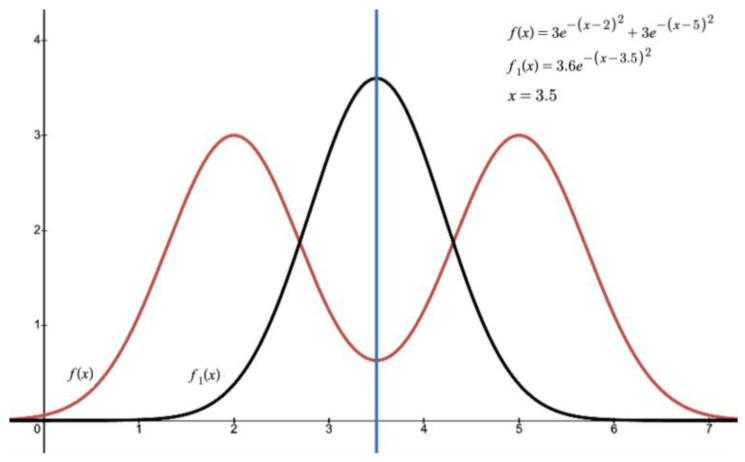
The red line (corresponding to the function *f*(*x*)) represents a bimodal distribution, suggesting two subgroups within the population, each with a different response profile or characteristic. In contrast, the black line, denoted as *f*_1_(*x*), illustrates a unimodal, normal (Gaussian) distribution. This distribution reflects a homogeneous response pattern with values clustering symmetrically around the mean. The blue vertical line marks the shared mean of the two functions, underscoring a key limitation: while mean is common to both distributions, it fails to capture the unique characteristics of each distribution.

Figure 2 (reproducible via the functions described in the above right) illustrates a bimodal distribution model in comparison to a normal distribution, highlighting the limitations of statistical analyses, when applied to abnormal distributions, in identifying clinically valuable data.

### 4.2. Metabolic Profiling Drugs: Metabolic Markers

The most accessible methods for studying cellular metabolism involve measuring metabolic products in blood samples or, when feasible, in the pericellular space. In certain contexts, radioisotopes can be employed to trace metabolic pathways [87] and provide detailed insights into cellular biochemical dynamics, although their use is typically restricted to preclinical research or specialized laboratory settings due to safety and regulatory limitations. Additional methods, such as advanced imaging techniques and fluorescence-based assays, can also provide valuable insights into metabolic activity and drug metabolism [88,89].

A more comprehensive and systems-level approach to studying metabolism is fluxomics, an emerging field that aims to analyze the rates of metabolic reactions within a biological system. Unlike traditional metabolic profiling, which provides a snapshot of metabolite concentrations at a given moment, fluxomics integrates data from multiple -omics fields (e.g., genomics, proteomics, and microbiomics) to quantify metabolic fluxes (the actual rates at which metabolites are produced and consumed within cells) [90]. This dynamic perspective allows researchers to understand how metabolic pathways are regulated in response to physiological or pathological conditions, providing deeper insights into disease mechanisms and potential therapeutic targets.

By combining fluxomics with other metabolic analysis methods, researchers can build a comprehensive toolkit for assessing individual metabolic status, gaining valuable insights into intracellular activity and metabolic abnormalities linked to various diseases.

At the core of these metabolic studies lies the metabolome, which represents the totality of the small molecules produced by metabolic processes. The metabolome constitutes a promising resource in medical research [91,92,93], providing information about the metabolic status of a subject and enabling the understanding of the metabolic abnormalities associated with various pathologies, which can be used to customize treatment.

The metabolome also serves as a mirror of intracellular activity, reflecting changes that occur following the administration of a drug and providing an effective means of studying individual metabolic responses. Additionally, prior to drug administration, biologically inert substances that produce no effects in the body but allow for the evaluation of individual metabolic characteristics through the measurement of metabolic by-products could be utilized.

**Figure 3:**
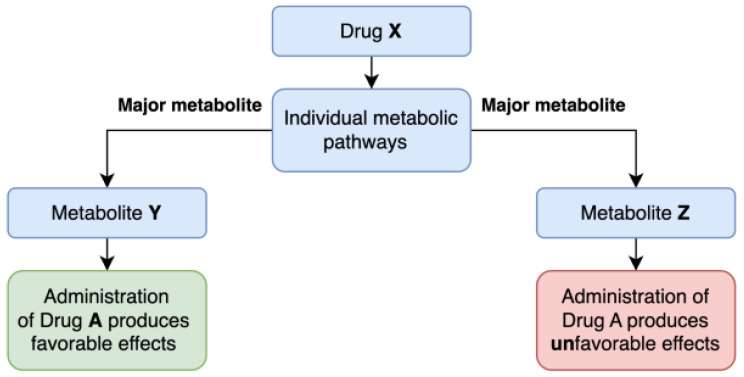
This diagram illustrates how individual metabolic variations, represented by their metabolic products (the primary metabolites Y and Z) resulting from the administration of Drug X, could predict a patient’s response to the subsequent administration of another drug. Consequently, the administration of Drug A may lead to different effects, either favorable or unfavorable, depending on the predominant metabolite.

We could refer to these substances as metabolic profiling drugs. By using them, we could identify population subgroups that exhibit common metabolic patterns, subgroups which could later be selected for clinical studies adapted to their metabolic profile. In this way, conclusions could be drawn about the effects of a drug by referring to the metabolic response that the population subgroup presents to the administration of the profiling drug, using a process that is illustrated in Figure 3.

An example of a drug that could be used for metabolic profiling is codeine. Codeine (methylmorphine) is a medication used to treat cough and pain. Its analgesic effect is primarily due to its conversion to morphine in the liver, a process mediated by the enzyme CYP2D6. This enzyme catalyzes the demethylation process, converting the methoxy group at position three of the aromatic ring into a hydroxyl group, resulting in morphine. Another metabolic pathway involves the enzyme CYP3A4, which transforms codeine by removing the methyl group from the nitrogen atom in codeine’s structure, producing norcodeine, an inactive metabolite in terms of analgesic effect. If CYP3A4 activity is predominant, less morphine is generated, leading to a weaker analgesic effect. Conversely, if CYP2D6 activity is dominant, more morphine is produced. This variability can result in significant heterogeneity in codeine’s effects, depending on individual metabolic characteristics.

CYP2D6 and CYP3A4 are part of the cytochrome P450 enzyme family, which is widely distributed across living organisms. These two enzymes play a role in the metabolism of a broad range of drugs [94,95,96], and differences in their activity contribute to the variability in individual responses to a drug. Codeine’s role as a metabolic profiler is particularly important for drugs that are substrates for both CYP2D6 and CYP3A4, as identifying the predominance of one enzyme’s activity can significantly aid in treatment personalization. Enzyme activity is subject to multiple variations, and this must be interpreted in the context of the specific substrate, as activity depends on affinity, which describes the enzyme–substrate interaction.

It is important to acknowledge the limitations of this approach, as each enzyme has distinct affinities for different chemical structures. Therefore, a patient with predominant CYP2D6 activity for one drug may still experience more intensive metabolism via CYP3A4 for another drug, depending on the chemical structure of the drug. To address this challenge and provide a more comprehensive view of overall metabolism, it may be necessary for a metabolic profiling drug to exhibit enzyme polyspecificity, allowing metabolic variations to be correlated more accurately with changes in metabolite concentrations.

## 5. Conclusions

One of the key conclusions of this review is the need to adapt clinical trial designs for drugs with highly variable reported effects. Stratifying participants based on their metabolic characteristics could help uncover strong correlations between specific factors and treatment outcomes, increasing this study’s relevance despite the heterogeneity of the observed effects across populational subgroups.

Large-scale studies have confirmed variability in cognitive outcomes associated with metformin use. A shift toward targeted, pathway-specific investigations may yield deeper insights into the biochemical mechanisms underlying these differences. Shifting from broad, population-based studies to precision-driven research can help optimize resource allocation and support the development of more personalized strategies for diabetes management and neuroprotection.

Given the important role that metabolic profiling drugs could play in personalized medicine, we propose that future research should consider biovariance-driven study designs, incorporating specific metabolic markers to enhance patient stratification. Additionally, the development of metabolic profiling drugs could enable more individualized treatments, improving both therapeutic efficacy and predictability.

These insights extend beyond metformin, providing a broader framework for understanding interindividual variability in drug responses and paving the way for more precise and effective therapeutic strategies.

## 6. Limitations

This review has several limitations that may affect the interpretation of findings. First, the selection of studies in this review is limited due to our selection criteria, database limitations, or the exclusion of publications in languages other than English. Second, the heterogeneity of the cognitive tests used in the analyzed studies complicates direct comparisons, as varied assessments may differ in their sensitivity to metformin’s cognitive effects. Additionally, linguistic and cultural differences in test adaptations may introduce biases, affecting cross-study consistency.

The new concepts proposed to explain the effects of metformin, like biovariance, have not been validated through experimental or clinical studies, highlighting the need for further research to confirm and apply these ideas in practice. The interpretation of data and development of these concepts may be influenced by our own perspectives, introducing a potential subjective bias on the part of the authors.

## Figures and Tables

**Table 1:** Summary of key studies addressing metformin’s effects.

Study	Objective	Methodology	Conclusion
Li et al. [17]	Evaluate the relationship between metformin use, cognitive impairment, and cerebral small vessel disease (CSVD) in type 2 diabetes patients	Cross-sectional study of patients with type 2 diabetes	Long-term metformin use was associated with reduced cognitive decline
Samaras et al. [18]	Evaluate if metformin use in older adults with type 2 diabetes is linked to a slower cognitive decline and lower incidence of dementia.	Prospective study comparing metformin users to non-users and non-diabetics over 6 years	Metformin users showed significantly slower decline in global cognition and executive function compared to non-users. Metformin also reduced dementia risk compared to non-users
Zhang et al. [19]	Assess the association between metformin therapy and cognitive dysfunction in type 2 diabetes patients	Meta-analysis of observational studies	Metformin therapy is linked to a reduced risk of neurodegenerative diseases, although effects vary based on treatment duration
Abbaszadeh et al. [20]	Examine metformin’s effect on white matter microstructural integrity in non-demented diabetic individuals	Study of non-demented diabetic subjects, divided into metformin users and non-users	Metformin users showed higher fractional anisotropy (FA) in the left hippocampal cingulum and right internal capsule, suggesting reduced neurodegeneration compared to non-users.
Rosell-Díaz et al. [21]	Explore the impact of metformin on cognitive function and gut microbiome in T2DM	Review of human studies, focusing on microbiome changes, cognitive function, and metabolic implications related to metformin	Metformin was shown to partially restore gut dysbiosis related to diabetes and may reduce dementia risk, although study results were not entirely consistent.
Tang et al. [22]	Assess dementia risk among veterans with type 2 diabetes using different drugs, including metformin	Observational study. Dementia risk comparison among those on metformin, sulfonylurea (SU), or thiazolidinedione (TZD)	Metformin alone had a moderate protective effect against dementia
He et al. [23]	Examine metformin’s impact on white matter integrity and cognitive impairment under chronic hypoperfusion conditions	Mouse model of chronic cerebral hypoperfusion	Metformin reduced white matter damage and improved cognitive function by preserving oligodendrocyte function
Newby et al. [24]	Assess the dementia and Parkinson’s disease risks in older adults with type 2 diabetes (metformin compared to sulfonylureas)	Study on metformin users and sulfonylurea users over a 5-year follow-up	MET users had a lower risk of all-cause dementia, Alzheimer’s disease, and vascular dementia compared to SU users. No significant difference for Parkinson’s disease.
Malazy et al. [25]	Assess the relationship between metformin therapy and cognitive performance	Systematic review and meta-analysis of 19 studies	Metformin therapy showed no significant improvement in cognitive function or protection against dementia
Huang et al. [26]	Examine the association between metformin use and dementia risk in patients with T2DM	Population-based study with 3- and 5-year follow-ups, categorizing patients by cumulative defined daily dose of metformin	Low-intensity metformin use was associated with a reduced dementia risk, while higher doses showed no protective effect.
Hui et al. [27]	Evaluate the effects of metformin on dementia, anxiety, and depression risks in diabetic patients	Retrospective cohort study	Metformin use was linked to significantly reduced risks of dementia, anxiety, and depression
Tang et al. [28]	Evaluate varied effects of metformin on dementia risk in T2DM patients	Longitudinal study on participants aged ≥50 with normal cognition at baseline	Metformin was linked to a reduced overall dementia risk, with varied effects across subgroups
Defo et al. [29]	Study the impact of diabetes and antidiabetic drugs on dementia risk	Systematic review and meta-analysis of 100 reviews and 27 cohort/case–control studies	MET, TZD, pioglitazone, GLP1 receptor agonists, and SGLT2 inhibitors significantly reduced dementia risk, particularly in Western populations.
Doran et al. [30]	Assess the dementia and mild cognitive impairment risks in T2DM patients using metformin versus other oral hypoglycemic drugs	Observational cohort study using UK primary healthcare records	Metformin use was associated with a 14% lower risk of dementia, with a more pronounced effect in patients under 80
Teng et al. [17]	Investigate metformin’s effects on cognitive impairment and cerebral small vessel disease (CSVD) in patients with type 2 diabetes	Case–control study	Metformin was associated with reduced cognitive impairment risk
Xue et al. [31]	Study the link between metformin use and severe dementia risk in Alzheimer’s patients with T2DM	Cohort study. Compared metformin users and non-users over 3.6 years	No significant association between metformin use and reduced severe dementia risk
Cho et al. [32]	Evaluate effects of long-term metformin treatment on cognitive function in Alzheimer’s model mice	The study examines the cognitive domains in transgenic and non-transgenic mice after 1 and 2 years of metformin treatment.	Metformin enhanced cognition in younger mice but impaired learning and memory in older AD model mice, increasing amyloid pathology and tau protein phosphorylation
Rabieipoor et al. [33]	Evaluate the therapeutic effects of metformin in a Alzheimer’s mice model	Alzheimer’s disease induced in mice using streptozotocin; treated with metformin, assessed with memory/cognitive tests and histopathological analysis	Metformin reduced neuroinflammation, preserved neuron integrity, and improved memory in Alzheimer’s model mice
Ping et al. [34]	Evaluate the effect of metformin on the incidence of neurodegenerative diseases (NDs), including dementia and Parkinson disease	Systematic review and meta-analysis of 19 observational studies with 285,966 participants	Metformin showed no significant protective effect on overall NDs. However, metformin monotherapy was linked to an increased Parkinson’s risk compared to non-metformin
Baradaran et al. [35]	Investigate the protective effects of metformin on memory impairment and oxidative stress caused by chronic ethanol exposure in adolescent rats	Adolescent rats were given ethanol with varying doses of metformin; memory and biochemical markers were assessed	Metformin reduced oxidative stress and neuroinflammation, preserving memory function in ethanol-exposed rats
Zheng et al. [31]	Compare dementia risk in T2DM patients treated with metformin versus those untreated	Observational cohort study	MET users showed a 12% lower dementia risk compared to untreated patients, with long-term users experiencing the greatest reduction
Shao et al. [36]	Investigate metformin’s effects on cognitive impairment and brain connectivity in patients with schizophrenia	Open-label, evaluator-blinded study on 72 schizophrenia patients randomized to metformin plus antipsychotics or antipsychotics alone	Metformin combined with antipsychotics improved cognitive scores significantly
Alnaaim et al. [37]	Investigate metformin’s anti-seizure effects and underlying mechanisms	Review study analyzing metformin’s influence on pathways like 5′ adenosine monophosphate-activated protein kinase (AMPK) and mTOR	Metformin exerts anti-seizure effects by activating AMPK and inhibiting mTOR, promoting neuroprotection via Brain-Derived Neurotrophic Factor (BDNF) expression and reducing inflammation
Singh et al. [38]	Evaluate metformin’s potential in epilepsy treatment	Systematic review of preclinical and clinical evidence	Metformin shows anticonvulsant effects by activating AMPK, inhibiting mTOR, protecting the blood–brain barrier, and reducing oxidative stress
Ha et al. [39]	Investigate the relationship between metformin use and Alzheimer’s disease risk in newly diagnosed T2DM patients	Retrospective, case–control study of dementia-free type 2 diabetes patients from Korea’s National Health Insurance database	Metformin use was associated with an increased risk of AD, particularly in patients with longer diabetes duration and concurrent depression
Paridari et al. [40]	Evaluate the impact of metformin on stroke risk in T2DM patients	Systematic review and meta-analysis of 21 studies, comparing stroke risk in metformin users versus other treatments	Metformin monotherapy was associated with a 34% reduction in stroke risk in randomized and cohort studies;
Akhtar et al. [41]	Evaluate the impact of chronic metformin use on stroke severity, outcome, and mortality in diabetic stroke patients	Analysis of acute stroke patients from Qatar; outcomes were compared between diabetic metformin users and non-users	Diabetic patients on metformin had significantly improved 90-day functional outcomes and lower mortality compared to those on other hypoglycemics
Tu et al. [42]	Assess the impact of metformin on fatality and disability rates post-stroke in T2DM patients	Cohort study of 7587 first-ever stroke patients in China, comparing metformin users vs. non-users over a 1-year follow-up	Metformin users had significantly lower in-hospital fatality rates and 12 month disability rates

**Table 2:** Intracellular targets of metformin.

Ref.	Hypothesized Cellular Target	Biological Function	Metformin Action
Zhang et al. [75] Li et al. [76]	AMPK	AMPK is a crucial kinase for cellular energy homeostasis, acting as a sensor of cellular energy status. Under low-energy conditions, AMPK activates pathways that promote ATP production and suppresses anabolic processes to conserve energy.	Metformin activates AMPK in neuronal tissues, potentially providing neuroprotective effects by promoting autophagy, reducing oxidative stress, and supporting mitochondrial function.
Apostolova et al. [77]	Glycerol-3-phosphate dehydrogenase 2 (GPD2)	GPD2 is a mitochondrial enzyme involved in redox transfer between the cytoplasm and mitochondria, particularly within the glycerophosphate shuttle.	Metformin modulates GPD2 activity indirectly through mitochondrial effects, promoting efficient glucose use.
Martín-Rodríguez et al. [78] Feng et al. [79]	Complex I	Complex I, also known as NADH oxidoreductase, is a key component of the mitochondrial electron transport chain (ETC).	Metformin inhibits Complex I, leading to controlled reductions in ATP, which activates AMPK. In neurons, this may promote protective autophagy, reduce oxidative damage, and improve mitochondrial efficiency
Guo et al. [80]	Forkhead box protein O1 (FOXO1)	FOXO1 is a transcription factor that regulates the expression of genes in many types of tissues, playing a central role in glucose homeostasis.	Metformin reduces FOXO1 activity by activating AMPK, which may limit neuronal apoptosis and enhance neurogenesis
Cao et al. [81]	Brain-derived neurotrophic factor (BDNF)	BDNF is a key neurotrophin essential for brain health, known for its role in promoting the growth, survival, and differentiation of neurons.	Metformin has been found to increase BDNF levels, supporting neurogenesis, synaptic plasticity, and potentially offering cognitive protection against neurodegenerative diseases.
Van Nostrand et al. [82]	Mammalian target of rapamycin complex 1 (mTORC1)	mTORC1 is a protein complex that regulates cell growth, protein synthesis, and nutrient sensing. It integrates signals related to nutrient availability, energy levels, and growth factors	Metformin inhibits mTORC1 activity, which may enhance autophagy in neurons and reduce pathological protein accumulation

## Data Availability

All data are reported in the text.

## Supplementary Materials

The following supporting information can be downloaded at https://www.mdpi.com/article/10.3390/ijms26041783/s1.
